# Effect of anterior femoral cortical notch grade on postoperative function and complications during TKA surgery: A multicenter, retrospective study

**DOI:** 10.1515/med-2024-0932

**Published:** 2024-04-15

**Authors:** Zhaokai Jin, Zhengming Wang, Kuangying Xu, Jiahao Chu, Sicheng Xiang, Yi Tang, Rui Wang, Haotian Hua, Zhongyi Zhang, Peijian Tong, Shuaijie Lv

**Affiliations:** Orthopedic Department, The First Affiliated Hospital of Zhejiang Chinese Medical University (Zhejiang Provincial Hospital of Chinese Medicine), Hangzhou, Zhejiang, China; Orthopedic Department, Shi’s Center of Orthopedics and Traumatology, Shuguang Hospital Affiliated to Shanghai University of Traditional Chinese Medicine, Shanghai, China; Orthopedic Department, The Second Affiliated Hospital of Zhejiang Chinese Medical University (Xinhua Hospital of Zhejiang Province), Hangzhou, Zhejiang, China; Orthopedic Department, The Third Affiliated Hospital of Zhejiang Chinese Medical University, Hangzhou, Zhejiang, China; Orthopedic Department, Guanghua Hospital, Affiliated to Shanghai University of Traditional Chinese Medicine, Institute of Arthritis Research in Integrative Medicine, Shanghai Academy of Traditional Chinese Medicine, Shanghai 200052, China; Orthopedic Department, Zhejiang Chinese Medical University, Hangzhou, Zhejiang, China

**Keywords:** anterior femoral notching, knee, arthroplasty, function, complication

## Abstract

**Purpose:**

To explore the effect of AFN on knee function and complications in patients after TKA.

**Methods:**

We evaluated 150 patients undergoing unilateral TKA, specifically including 102 patients with varying degrees of AFN after selection. They were divided into four groups based on AFN grade. About 48 patients did not produce AFN, 63 patients were grade I, 29 patients were grade II, and 10 patients were grade III. All patients were followed up for 24 months, and knee function, pain, complications, and other indicators were compared between the four groups. Correlation analysis and regression analysis were used to study the relationship between AFN and other indicators.

**Results:**

Two cases of periprosthetic fractures (PPF) occurred in our study, with an incidence of 1.35%, which did not show a significant association with AFN. The changes in knee social score (ΔKSS), Western Ontario and McMaster Universities Osteoarthritis Index (ΔWOMAC), and postoperative anterior knee pain visual analog scale (VAS) score were higher in patients with AFN than in those without. Particularly, grades II and III AFN demonstrated superior efficacy. Pearson’s correlation analysis showed that AFN grade is positively correlated with both ΔKSS and ΔWOMAC (*r* = 0.44, *P* < 0.001), and AFN grade had a negative correlation with the anterior knee pain VAS (*r* = −0.250, *P* < 0.05). In linear regression analysis, AFN grade was positively correlated with both ΔKSS (*β* = 5.974, 95% CI: 3.968–7.981, *P* < 0.001) and ΔWOMAC (*β* = 6.356, 95% CI: 4.223–8.490, *P* < 0.001). Besides that, there was a negative correlation between AFN grade and anterior knee pain (*β* = 5.974, 95% CI: 3.968–7.981, *P* < 0.05).

**Conclusion:**

Patients with grade II and III AFN who underwent TKA exhibited better knee function and lower levels of anterior knee pain post-surgery.

## 1 Introduction

Total knee arthroplasty (TKA) is one of the common methods to deal with end-stage knee osteoarthritis (KOA) [1] and has been widely practiced clinically. Anterior femoral notching (AFN) is a common occurrence in TKA and its implications on postoperative satisfaction and outcomes are important to consider. AFN is possibly associated with the use of posterior reference systems, navigated TKA, femoral sizer design, or limited size options of femoral components in TKA systems [2]. The incidence of AFN, which involves artificial osteotomy of the anterior cortex of the distal femur for prosthesis placement, ranges from 3.5 to 26.9% [3]. According to the Gujarathi scale [4], AFN is divided into four grades: (1) Grade I: invasion of the anterior cortical surface of the femoral, (2) Grade II: invasion of the outer and inner surface of the femoral cortex, (3) Grade III: 25% invasion of the medullary canal (from the inner surface to the center of the medullary canal), and (4) Grade IV: 50% invasion of medullary canal (inner surface to center of medullary canal). Previous research [5,6] shows that AFN is associated with the occurrence of postoperative periprosthesis fracture (PPF), and the grade of AFN may have a certain impact on postoperative knee pain, anterior knee pain, and joint function. However, existing research still has different perspectives on the postoperative effects of AFN [5–8], and exploring the complex effects of AFN on TKA patients has far-reaching implications. Therefore, the aim of this retrospective multicenter study was to investigate the effects of different grades of AFN on postoperative knee function and complications in patients who underwent TKA.

## 2 Subjects and methods

### 2.1 Study design

This study was conducted at three orthopedic centers in Zhejiang Province and was approved by the ethical review committees of each institution. All patients signed informed consent forms before surgery, and all information was complete and available for publication. Patients who received an elective primary unilateral TKA at various medical centers between January 2018 and January 2021 were included in the study. After excluding patients with revision, long-term use of steroids and immunosuppressive drugs, and patients with hemophilia, rheumatoid KOA, and other diseases, a total of 150 patients were included in the study. All patients have been followed up for at least 24 months. Record and compare the basic indicators of all patients such as age, gender, body mass index (BMI), and Kellgren/Lawrence (K/L) [9] classification before surgery. The knee function, complications, and other indicators of the patient from preoperative to follow-up were recorded and used for analysis and comparison.

### 2.2 Radiographic examination

All patients underwent standard radiographs (AP view, lateral view, and long-leg standing radiographs) before and after TKA. X-ray images were used to measure and evaluate the depth of intraoperative AFN for grading. During the follow-up, regular X-ray reviews were conducted, and an immediate review of radiographs was performed if complications such as fractures occurred.

### 2.3 Surgical technique

General anesthesia was used for TKA, and a tourniquet was applied until skin closure. The surgical approach involved an anterior midline incision and a medial parapatellar approach. After determining the femoral bone marrow location, femoral varus osteotomy was performed followed by distal femur osteotomy using a “four-in-one” osteotomy guide with the femur’s anterior reference, and the tibial side osteotomy was completed by the extramedullary localization. Joint stability and patellar trajectory were assessed using a trial mold. Patellar replacement was not performed in any case. Finally, a cemented femur–tibia prosthesis was implanted, and the incision was sutured without routine placement of a drainage tube. Antibiotics and thrombosis prevention measures were administered postoperatively. Each patient’s surgery is performed by the same surgeon in their center. All patients received bone cement-stabilized (PS) knee prostheses provided by Stryker^®^ (Stryker Scrorpio NRG Knee-flexed) and Zimmer^®^ (NexGen LPS).

### 2.4 Postoperative management

A regular rehabilitation program was initiated on the day after surgery under the guidance of a professional rehabilitation trainer. This program included passive and active knee movements, quadriceps exercises, proprioception training, gait training, and functional retraining for activities of daily living. During regular follow-up, the visual analog scale (VAS) for pain, knee range of motion (ROM), knee social score (KSS) [10], Western Ontario and McMaster Universities Osteoarthritis Index (WOMAC) [11], and complications were documented.

### 2.5 Outcome measurement

#### 2.5.1 Knee joint function assessment

Knee function was evaluated by preoperative and postoperative changes in knee ROM (ΔROM), changes in KSS (ΔKSS), and changes in WOMAC (ΔWOMAC).

#### 2.5.2 Complications and satisfaction

Complications were recorded in all patients within 24 months of surgery. The pain at the patella and the incision site of the anterior approach surgery was defined as anterior knee pain and recorded using VAS. Likert scale was used for patient satisfaction [12], which was recorded as 1–5 points for statistical inclusion.

### 2.6 Statistical analysis

All data were analyzed with SPSS 25.0 (IBM, Armonk, NY, USA). The Kolmogorov–Smirnov test was used to judge the normality of the sample. One-way repeated-measure analysis of variance with Tukey–Kramer correction or Kruskal–Wallis test with Steel–Dwass correction was used to compare the results of preoperative and postoperative changes in various indicators in the four groups. In addition, we used linear regression to analyze the relationship between AFN grade and partial postoperative indicators. Pearson correlation analysis was used to evaluate the correlation between various indicators. *P* < 0.05 was considered statistically significant.

The calculation parameters for the sample were set as follows: the occurrence of PPF is 0.3–2.5% of all TKAs used to treat end-stage arthritis in the published literature [13]; alfa: 0.05; power: 80%. The loss of follow-up rate was 20%, and the minimum sample size obtained by this method was 124, so the sample size was set at 150 in this study.


**Ethical approval and consent to participate:** This retrospective study has been approved by the Institutional Review Committee of The First Affiliated Hospital of Zhejiang Chinese Medical University ethics committee (No. 2023-KLS-159-01), The Second Affiliated Hospital of Zhejiang Chinese Medical University ethics committee (No. 2023-LW-011-01), and The Third Affiliated Hospital of Zhejiang Chinese Medical University ethics committee (No. ZSLL-ZN-2023-008-01) without specific consent from patients.
**Work location declaration:** All participating work units have passed ethical approval for this study, approved the use of case data, and authorized this study. At the same time, all units and research participants declare that there are no conflicts of interest in this study.

## 3 Results

### 3.1 Participant characteristics

**Figure 1:**
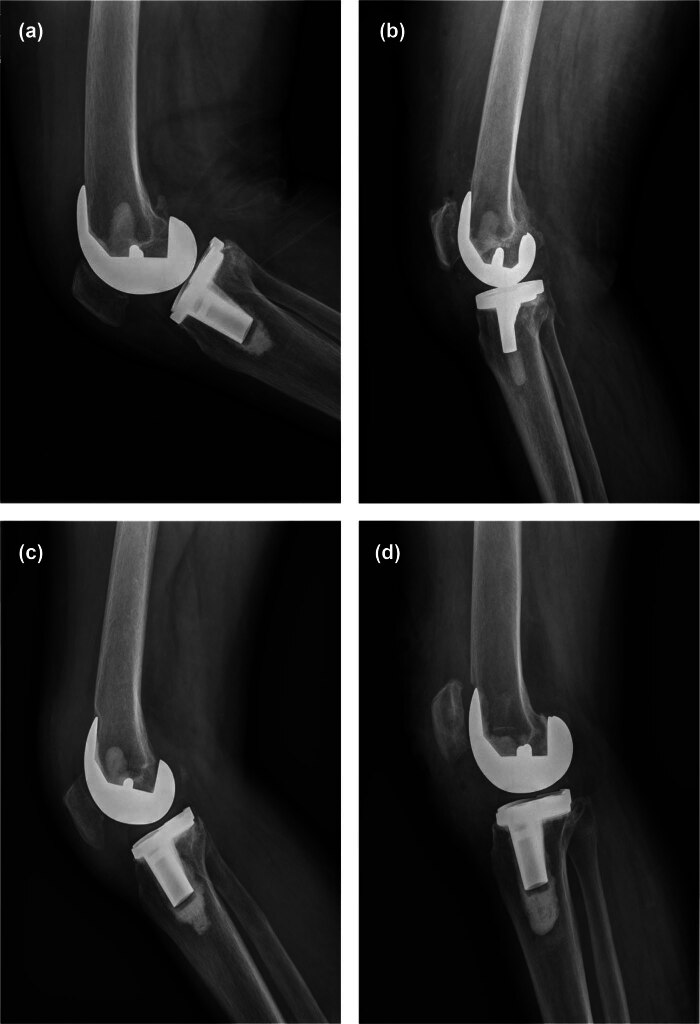
Patient examples from our study: (a) none AFN, (b) grade I AFN, (c) grade II AFN, and (d) grade III.

Among the patients included in this study, 50 (33.33%) were male patients. The mean age of patients in this series was 73.85 ± 7.60 years, the mean BMI was 25.28 ± 3.99, and the mean length of hospitalization was 25.64 ± 7.24 days (Table 1). According to the Gujarathi classification, 48 (32.00%) patients did not produce AFN, 63 (42.00%) patients were grade I (invasion of the anterior cortical surface of the femoral), and 29 (19.33%) patients were grade II (invasion of the outer and inner surface of the femoral cortex). Ten patients (6.67%) were grade III (25% invasion of the medullary canal), and no patients with grade IV AFN were found in this study (Figure 1). There was no significant difference in gender, BMI, and preoperative indicators among the patients in different grades (*P* > 0.05) (Table 1), and also no significant difference among the clinical centers (Table A2).

**Table 1:** General demographic and preoperative clinical characteristics of patients

Variables		AFN grade (Gujarathi taxonomy)				*P* value
	Total	None	Grade I	Grade II	Grade III	
	*N* = 150	*N =* 48	*N =* 63	*N =* 29	*N =* 10	
Age	73.85 ± 7.60	74.25 ± 6.83	73.89 ± 7.98	73.24 ± 8.27	73.40 ± 7.62	0.950
BMI	25.28 ± 3.99	24.50 ± 2.50	25.47 ± 4.34	25.92 ± 5.29	25.95 ± 2.91	0.396
Male (*n*/%)	50 (33.3)	19 (39.6)	15 (23.8)	13 (44.8)	3 (30.0)	0.161
Left (*n*/%)	80 (53.3)	25 (52.1)	34 (54.0)	16 (55.2)	5 (50.0)	0.989
K/L classification (*n*/%)	123 (82.0)	41 (85.4)	55 (87.3)	25 (86.2)	2 (80.0)	0.942
WOMAC	75.29 ± 10.44	72.08 ± 10.43	76.27 ± 10.87	78.07 ± 6.56	73.50 ± 11.80	0.053
KSS	40.00 ± 9.27	41.04 ± 7.22	41.11 ± 11.12	36.72 ± 8.16	37.50 ± 5.89	0.118
ROM (°)	79.83 ± 19.16	85.10 ± 21.33	79.05 ± 18.66	74.14 ± 10.09	83.00 ± 17.03	0.071
Anterior knee pain VAS	2.74 ± 1.10	2.90 ± 1.12	2.81 ± 1.13	2.52 ± 1.02	2.20 ± 0.92	0.187

BMI, body mass index; K/L classification, Kellgren/Lawrence classification; WOMAC, Western Ontario and McMaster universities osteoarthritis index; KSS, knee society score; ROM, range of motion; VAS, visual analog scale.

### 3.2 Effect of AFN grade on knee joint function and complications

Patients showed significant improvement in knee joint function and other indicators after TKA surgery (Table A1). However, as shown in Table 2, in terms of ΔKSS, the AFN group 30.98 ± 12.55 was significantly higher than the non-AFN group 19.67 ± 10.48 (*P* < 0.05). In Table 3, the group with the greatest improvement was 37.76 ± 10.74 in grade II, followed by 31.00 ± 6.58 in grade III (*P* < 0.05). In ΔWOMAC, 38.04 ± 11.34 in AFN group was higher than 28.75 ± 11.78 in non-AFN group (*P* < 0.05) (Table 2). The biggest change was also 43.24 ± 7.28 in grade II, followed by grade III 43.00 ± 8.31 (*P* < 0.05) (Table 3). Postoperative anterior knee pain VAS in the AFN group was lower than that in the non-AFN group (*P* < 0.05) (Table 2), and the patients with grade II 0.55 ± 0.95 and grade III 0.50 ± 0.85 AFN were lower than other AFN patients (*P* < 0.05) (Table 3).

**Table 2:** Postoperative knee joint function and pain assessment of non-AFN and AFN group

Variable	Non-AFN (*N* = 48)	AFN (*N* = 102)	*P* value
ΔWOMAC	28.75 ± 11.78	38.04 ± 11.34	0.000
ΔKSS	19.67 ± 10.48	30.98 ± 12.55	0.000
ΔROM (°)	23.54 ± 14.62	23.19 ± 14.45	0.889
Anterior knee pain VAS	1.25 ± 1.12	0.85 ± 1.01	0.032
ROM (°) maximum	104.06 ± 13.43	101.42 ± 12.75	0.247

AFN, anterior femoral notching; ΔWOMAC, changes of the Western Ontario and McMaster universities osteoarthritis index; ΔKSS, changes of the knee society score; ΔROM, changes of the range of motion; VAS, visual analog scale.

**Table 3:** Postoperative knee joint function and pain assessment in each AFN group

Variable	AFN grade (Gujarathi taxonomy)			
	None (*N* = 48)	Grade I (*N* = 63)	Grade II (*N* = 29)	Grade III (*N* = 10)
ΔWOMAC	28.75 ± 11.78^a^	34.86 ± 12.18^a^	43.24 ± 7.28^b^	43.00 ± 8.31^c^
ΔKSS	19.67±10.48^a^	27.86±12.91^ab^	37.76±10.74^b^	31.00±6.58^c^
ΔROM (°)	23.54 ± 14.62^a^	22.06 ± 14.50^a^	26.03 ± 14.84^a^	22.00 ± 13.17^a^
Anterior knee pain VAS	1.25 ± 1.12^a^	1.05 ± 1.02^a^	0.55 ± 0.95^b^	0.50 ± 0.85^b^
ROM (°) maximum	104.06 ± 13.43^a^	101.90 ± 12.20^a^	99.14 ± 14.21^a^	105.00 ± 11.79^a^

AFN, anterior femoral notching; ΔWOMAC, changes of the Western Ontario and McMaster universities osteoarthritis index; ΔKSS, changes of the knee society score; ΔROM, changes of the range of motion; VAS, visual analog scale. Values with superscript letter a, b, c, and d are significantly different across columns (*P* ＜ 0.05).

### 3.3 Correlation analysis

As shown in Table 4, Pearson’s correlation analysis showed that the AFN grade was significantly correlated with both the ΔKSS and ΔWOMAC, and they are all positively correlated (*r* = 0.44, *P* < 0.001). AFN grade had a significant negative correlation with the anterior knee pain VAS (*r* = −0.250, *P* < 0.05). And there is no significant correlation between AFN grade and other included indicators.

**Table 4:** Pearson correlation analysis of main outcomes, AFN grade and other indicators

Variables	AFN grade	
	*r*	*P*
Age	−0.046	0.579
BMI	0.131	0.109
Sex	−0.005	0.948
Clinical center	0.000	1.000
ΔWOMAC	0.433	0.000
ΔKSS	0.433	0.000
Anterior knee pain VAS	−0.250	0.002

AFN, anterior femoral notching; ΔWOMAC, changes of the Western Ontario and McMaster universities osteoarthritis index; ΔKSS, changes of the knee society score; VAS, visual analog scale.

### 3.4 Linear regression analysis

Linear regression analysis in Table 5 shows that, in our unadjusted analysis, AFN grade is positively correlated with both ΔKSS (*β* = 5.974, 95% CI: 3.968–7.981, *P* < 0.001) and ΔWOMAC (*β* = 6.356, 95% CI: 4.223–8.490, *P* < 0.001). Besides that, there was a negative correlation between AFN grade and anterior knee pain (*β* = 5.974, 95% CI: 3.968–7.981, *P* < 0.05) (Table 6). After further adjusting our analysis for the covariates, there is still a significant correlation between AFN and the above three indicators.

**Table 5:** Linear regression analysis of AFN grade and knee function

Variables	ΔWOMAC				ΔKSS			
	*β* (95% CI)	*P*	*β* (95% CI)*	P*	*β* (95% CI)	*P*	*β* (95% CI)*	P*
AFN grade	5.974 (3.968–7.981)	0.000	5.915 (3.907–7.922)	0.000	6.356 (4.223–8.490)	0.000	6.221 (4.060–8.381)	0.000

AFN, anterior femoral notching; ΔWOMAC, changes of the Western Ontario and McMaster universities osteoarthritis index; ΔKSS, changes of the knee society score; *β*, *β*-coefficient; CI, confidence interval; *Linear regression analysis after adjusting age (years), sex (men or women), BMI (kg/m^2^), clinical center and other covariates.

**Table 6:** Linear regression analysis of AFN grade and anterior knee pain

Variables	Anterior knee pain VAS			
	*β* (95% CI)	*P*	*β* (95% CI)*	P*
AFN grade	−0.299 (−0.485 to −0.113)	0.002	−0.291 (−0.480 to −0.102)	0.003

AFN, anterior femoral notching; VAS, visual analog scale. *β*, *β*-coefficient; CI, confidence interval; *Linear regression analysis after adjusting age (years), sex (men or women), BMI (kg/m^2^), clinical center and other covariates.

## 4 Discussion

TKA is a widely used surgical option to treat end-stage KOA or other serious knee diseases [14]. During primary TKA, different grades of AFN may occur [15]. Many studies prove that [5,7] AFN also has an impact on postoperative knee ROM and knee. Junya et al. [5] concluded that overfilling the knee joint after TKA can lead to decreased ROM and pain in the anterior knee area. There was no significant effect on knee pain. However, some studies [8,16] stated that there was no significant association between AFN and KSS of knee motion. We found that AFN grade was negatively correlated with the probability and grade of anterior knee pain within a certain range. Additionally, ΔWOMAC and ΔKSS were positively correlated with AFN grade, reaching the maximum in patients with grade II and grade III AFN. We believe that one of the reasons for this phenomenon may be due to the physiological characteristics of different populations and races affecting the measurement of joint morphology [17]. Many studies [18–20] have compared the structure of the Asian knee (Japanese, Chinese, Indian, and Korean) to existing Western-designed total knee implant systems. These anthropometric studies have suggested that Western-designed knee prostheses may not be suitable for Asian knees. The immediate effect after surgery is that the oversized prosthesis leads to overfilling of the patient’s relatively small patellofemoral joint, which increases discomfort and limited ROM in the anterior knee area [21]. However, the surgical goal is not only to ensure proper anchoring of the implant in the anterior femoral cortex but also to avoid overfilling of the patellofemoral cavity [22]. In our study, most of the patients we selected developed AFN due to a lack of standardization in surgical procedures. However, there are also some patients whose flexion gap is larger than the extension gap. In order to balance the flexion and extension gap, we will move the “four in one” osteotomy guide plate back 1–2 mm, artificially causing AFN to achieve posterior femoral offset and ultimately achieving the goal of balancing the flexion extension gap. Cheng et al. [23] found that existing prostheses of different sizes have varying degrees of oversize on the anterior or lateral sides of the tibia after implantation in Asian patients. Therefore, some scholars advocate for appropriate osteotomy of the anterior femoral cortex to reduce anterior knee pain and limited motion caused by overfilled knee joints, such as the suggestion from Antinolf [7] to use moderate AFN for backward implantation of the prosthesis, aligning it with the anterior femoral cortex. This approach can lead to better postoperative functional outcomes and effectively reduce anterior knee pain, which is partly consistent with the results of our study.

Moreover, most of the existing research suggests that [6,21] excessive AFN may increase the incidence of postoperative PPF. Culp et al. [6] studied 61 patients with fractures, and 27 had significant gaps. Biomechanical analysis was used to set the threshold at 3 mm, and AFN greater than this depth would lead to a significant reduction in femoral anti-torsional strength and increase the risk of PPF. The studies of Shekhar et al. [21] and Madsen et al. [24] also suggested that the appropriate prosthesis for patients should be identified in various ways during TKA surgery to reduce the occurrence of AFN, so as to avoid the occurrence of PPF. However, some scholars have a different opinion, suggesting that AFN has no significant relationship with knee function, pain, and PPF after TKA. Gujarathi et al. [4] and some other research [22] concluded that bone remodeling around the defect area after AFN reduced the risk of postoperative fracture in patients with AFN. In our study, there is no occurrence of deep venous thrombosis, infection, or skin ulcers, which is related to our meticulous perioperative management. Only in one patient with grade I AFN and one patient with grade II AFN PPF occurred due to fall 6 months after surgery. Both cases had supracondylar fractures and returned to normal after undergoing secondary revision, resulting in an overall PPF occurrence rate of only 1.35%. This is consistent with the results reported in some existing literature. According to Ritter et al. [8] the incidence of distal femoral fracture was approximately 0.5% in 27% of postoperative TKA patients AFN. In a report by José et al. [25], it was found that there was no significant correlation between supracondylar fracture and AFN in a control group of 50 patients with supracondylar fracture and 100 patients without supracondylar fracture after TKA surgery. Similarly, our results show that Gujarathi I–III AFN does not increase the incidence of PPF. In summary, some current studies tend to suggest that AFN can lead to a decrease in the strength of the distal femur and an increase in the incidence of PPF [6,21,24]. At the same time, some studies suggest that according to Wolff’s law, AFN can cause the surrounding bone cortex to self-rebuild and form compensatory bone reinforcement, so AFN does not significantly increase the incidence of PPF [4,22]. And by releasing the pressure in the front of the joint, the knee joint can achieve a better ROM and function after surgery [7]. Our research results seem to lean more toward the latter. However, we still insist on treating the results obtained in this study with caution, and the impact of AFN on postoperative TKA patients is still a controversial point that needs further exploration. The mechanism of its impact on different patients in different situations is complex. Our research findings attempt to provide some new findings and research ideas for exploring the impact of AFN grade on postoperative function and complications in TKA.

Our study has several limitations. First of all, factors influencing knee joint function and pain after TKA are diverse and complex, and this study did not completely exclude all interfering factors except AFN, resulting in unavoidable deviations in experimental results. Second, our study is a retrospective study that is more prone to bias and error, which has a certain grade of influence on the authenticity of the experiment. Finally, we used fewer types of prostheses and did not rule out the impact of different types of prostheses on the study.

## 5 Conclusion

The patients who have experienced Gujarathi II and III AFN in TKA have less pain in the anterior area of the knee joint and their joint function is relatively better. Clearly, larger samples and longer follow-up are needed to enhance the applicability of this conclusion and further understand the complex relationship between AFN and postoperative complications.

## Appendix

**Table A1:** Preoperative General demographic and clinical characteristics of patients in each hospital

Variable	Clinical Center			*P* value
	Center 1	Center 2	Center 3	
	*N* = 49	*N* = 52	*N* = 49	
Age	72.33 ± 8.11	74.81 ± 7.05	74.65 ± 6.95	0.183
BMI	26.21 ± 3.26	24.95 ± 3.16	25.39 ± 2.46	0.105
Male (*n*/%)	15 (30.6%)	16 (30.8%)	19 (38.8%)	0.618
Left (*n*/%)	23 (46.9%)	30 (57.7%)	27 (55.1%)	0.534
WOMAC	77.86 ± 9.02	73.40 ± 9.60	74.63 ± 11.06	0.071
KSS	38.98 ± 8.04	40.38 ± 10.04	40.61 ± 9.66	0.641
ROM (°)	79.29 ± 12.20	83.94 ± 12.46	76.02 ± 28.15	0.112
Anterior knee pain VAS	2.47 ± 0.89	2.44 ± 0.83	2.16 ± 0.87	0.153
ROM (°) Maximum	101.43 ± 13.07	102.69 ± 12.06	102.65 ± 14.04	0.861

AFN, anterior femoral notching; WOMAC, Western Ontario and McMaster universities osteoarthritis index; KSS, knee society score; ROM, range of motion; VAS, visual analogue scale; BMI, body mass index.

**Table A2:** Comparison of main indicators in preoperative and postoperative

Variable	Preoperative	Postoperative	*t* value	*P* value
	Mean ± SD	Mean ± SD		
WOMAC	75.09 ± 10.26	40.31 ± 11.42	30.939	0.000
KSS	40.00 ± 9.27	67.46 ± 10.84	−25.762	0.000
ROM (°)	80.30 ± 18.47	102.27 ± 12.99	−15.604	0.000
Anterior knee pain VAS	2.36 ± 0.87	0.98 ± 1.06	15.761	0.000

WOMAC, Western Ontario and McMaster universities osteoarthritis index; KSS, knee society score; ROM, range of motion; VAS, visual analogue scale.

**Table A3:** Postoperative complications and satisfaction data of patients in each group

Variable	
Complications	
Periprosthetic	2/1.33%
fractures	
Infection	0/0
DVT	0/0
Skin ulceration	0/0
Satisfaction	150/100%
Very satisfed	94/62.27%
Satisfed	52/34.67%
Disappointed	4/2.67%
Dissatisfed	0/0
Neutral	0/0

DVT, deep venous thrombosis.
